# Does journal endorsement of reporting guidelines influence the completeness of reporting of health research? A systematic review protocol

**DOI:** 10.1186/2046-4053-1-24

**Published:** 2012-05-24

**Authors:** Larissa Shamseer, Adrienne Stevens, Becky Skidmore, Lucy Turner, Douglas G Altman, Allison Hirst, John Hoey, Anita Palepu, Iveta Simera, Kenneth Schulz, David Moher

**Affiliations:** 1 Centre for Practice-Changing Research, Ottawa Hospital Research Institute, The Ottawa Hospital – General Campus, 501 Smyth Road, Box 201B, Ottawa, ON, K1H 8L6, Canada; 2 Independent Research and Information Consultant, Ottawa, ON, Canada; 3 Centre for Statistics in Medicine, University of Oxford, Oxford, UK; 4 Queen’s University, Kingston, ON, Canada; 5 St. Paul’s Hospital, Vancouver, BC, Canada; 6 FHI 360, Durham, NC, USA

**Keywords:** Reporting guidelines, Evaluation, Systematic review, Completeness of reporting

## Abstract

**Background:**

Reporting of health research is often inadequate and incomplete. Complete and transparent reporting is imperative to enable readers to assess the validity of research findings for use in healthcare and policy decision-making. To this end, many guidelines, aimed at improving the quality of health research reports, have been developed for reporting a variety of research types. Despite efforts, many reporting guidelines are underused. In order to increase their uptake, evidence of their effectiveness is important and will provide authors, peer reviewers and editors with an important resource for use and implementation of pertinent guidance. The objective of this study was to assess whether endorsement of reporting guidelines by journals influences the completeness of reporting of health studies.

**Methods:**

Guidelines providing a minimum set of items to guide authors in reporting a specific type of research, developed with explicit methodology, and using a consensus process will be identified from an earlier systematic review and from the EQUATOR (Enhancing the QUAlity and Transparency Of health Research) Network’s reporting guidelines library. MEDLINE, EMBASE, the Cochrane Methodology Register and Scopus will be searched for evaluations of those reporting guidelines; relevant evaluations from the recently conducted CONSORT systematic review will also be included. Single data extraction with 10% verification of study characteristics, 20% of outcomes and complete verification of aspects of study validity will be carried out. We will include evaluations of reporting guidelines that assess the completeness of reporting: (1) before and after journal endorsement, and/or (2) between endorsing and non-endorsing journals. For a given guideline, analyses will be conducted for individual and the total sum of items. When possible, standard, pooled effects with 99% confidence intervals using random effects models will be calculated.

**Discussion:**

Evidence on which guidelines have been evaluated and which are associated with improved completeness of reporting is important for various stakeholders, including editors who consider which guidelines to endorse in their journal editorial policies.

## Background

### Reporting guidelines

Reporting of health research is, in general, inadequate [1-6]. Complete and transparent reporting is imperative to assessing the validity of reported treatment effects and other findings of health research. A study’s methods should be described in enough detail so they can be replicated, the analyses should follow the protocol, and the results should be given in full enough detail to be incorporated into future research. Complete and transparent reporting enables clinicians and others to make better, more informed health care decisions. Transparent reporting is an integral part of the research process and facilitates the interpretation of whether good science was employed. For instance, without a description of the methods used to control internal validity (for example, randomization, blinding) and external validity (for example, definition of the population under study), the reader is left to guess at whether the treatment effect estimate reported is accurate. To ameliorate the problem of inadequate reporting, many guidelines have been developed, aimed at improving the quality of reports of health research. A reporting guideline aids authors in the reporting of specific types of research and may be accompanied by a checklist of important items to be reported and, potentially, a flow diagram to describe the study process or explicit guidance text [7,8].

The EQUATOR (Enhancing the QUAlity of Transparency Of health Research) Network is an international initiative promoting transparent and accurate reporting of health-related research. As of October 2011, 191 reporting guidelines were indexed in the Library for Health Research Reporting on the EQUATOR website [9]. Of the 90 indexed in September 2009, 81 were included in a systematic review that characterized their development process. Guidelines in that review date from as early as 1986 but many are more recent; approximately half were developed between 2005 and 2009 [8]. Those numbers demonstrate considerable and increasing investment in the development of reporting guidelines.

### Importance for journal editors

Journals are the most important conduit for publishing health research. Some reporting guidelines have received positive attention, in the form of endorsement by health journals (for example, the CONsolidated Standards Of Reporting Trials (CONSORT) Statement is endorsed by over 600 journals). Such endorsement is typically evidenced by a statement in a journal’s “Instructions to Authors” regarding the use (suggested or required) of one or more guidelines while preparing a study manuscript. Some journals publish editorials indicating their support, while others institute mandatory submission of a guideline checklist and/or flow diagram along with manuscript submission.

Editors are constantly striving to ensure that what is published in their journals is, clear, complete, transparent, and as free from bias as possible. A recent survey indicates that almost half of journal editors who responded (n = 67) consider completeness of reporting to be one of the top three factors when making a publication decision [10]. In the same survey, however, 18% of the editors who were interviewed perceived endorsement of a reporting guideline as burdensome. Furthermore, in an effort to uphold high standards, journal editors may feel the need to endorse multiple reporting guidelines without knowledge of their rigor or ability to improve reporting. Evidence about their effect may provide editors with the rationale for making decisions about which to endorse at their journals.

### Evaluation of reporting guidelines

To date, there have been few evaluations of reporting guidelines. In many ways reporting guidelines are a checklist of important items that ought to be carried out. In other fields such checklists have proven to be of great benefit. For example, an evaluation of the World Health Organization (WHO) Surgical Safety Checklist showed that its implementation is associated with a 47% reduction in the rate of death and 36% reduction in inpatient complications after surgery [11]. Not only do these numbers demonstrate the usefulness of a checklist in improving mortality and morbidity in surgery, they are arguably the most fundamental piece of information needed to initiate change in surgical practice. Based on these numbers, implementation of the WHO checklist is taking place on a global scale. Similarly, checklists for reporting research must be evaluated in order to provide evidence for knowledge users to make informed decisions about their implementation.

With respect to reporting guideline evaluation, only 17% of guideline developers report an intention to formally evaluate their guidelines; 7% indicate an explicit intention *not* to evaluate their guideline post-publication [8]. In a 2008 survey of developers of 30 reporting guidelines, only 17% (n = 5) stated having formally evaluated the impact of their guidelines on the completeness of reporting of research for which it was intended [12]. These numbers demonstrate a gap between guideline development and quality control, leaving development efforts wasted if stakeholders are unable to judge their effectiveness and, as such, subsequently unable to endorse a guideline.

One reporting guideline that has been extensively evaluated is the CONSORT Statement [13-17]. In 2006, a systematic review of CONSORT effectiveness identified eight studies evaluating its impact [18]. A 2012 update of that review identified 42 additional evaluations [19,20]. Both found that endorsement of CONSORT by journals is significantly associated with a higher frequency of completely reported trials, at least for some items of the CONSORT Statement. Evaluations of other guidelines, such as the Standards for the Reporting of Diagnostic Accuracy (STARD) statement in 2003 [21] and the Standards for Reporting Interventions in Clinical Trials of Acupuncture (STRICTA) Statement [22], are known to exist but have not yet been systematically identified and synthesized. It may be that these examples indicate a fraction of evaluations that actually exist. Bringing together evaluations of reporting guidelines will provide a resource for knowledge users to aid in the decision-making process of whether or not to implement a reporting guideline, improve the uptake of reporting guidelines overall, and highlight areas of reporting still left to be evaluated.

## Objectives

The objective of this systematic review is to evaluate whether journal endorsement of reporting guidelines influences the completeness of the reported literature by comparing:

1. Completeness of reporting of studies published in journals endorsing reporting guidelines before and after endorsement.

2. Completeness of reporting of studies published in journals that have and have not endorsed reporting guidelines.

## Methods

### Criteria for including studies in this review

#### *Types of studies*

In order to identify evaluations of reporting guidelines, we first need to determine the set of reporting guidelines for which we will be seeking evaluations and subsequently, on which to base the search.

##### *Eligibility of reporting guidelines of interest*

Reporting guidelines for health research, including clinical, basic science and laboratory research, and written only in English, (for feasibility), will be included.

Eligibility criteria will be based on a recent systematic review that identified and characterized the reporting guideline process for 81 reporting guidelines for health research [8]. Potential reporting guidelines will be included if they contain explicit text to guide authors in the reporting of a specific type of research (which may or may not be accompanied by a checklist of reporting criteria and/or a flow diagram) and if they describe how the guideline was developed and how consensus among the developers was obtained. This criteria is intended to differentiate reporting guidelines from other efforts in which guidance was not developed using explicit methods, even if a checklist is produced, such as the recent guidance for reporting myeloma trials - described as ‘consensus’ in the title - but no methods of development or consensus were otherwise described [23]. Selection criteria will also exclude guidance on the formatting of reports of health research, such as found in some journals’ 'Instructions to Authors’. Reporting guidelines for purposes other than reporting research (for example, clinical case report forms) will also be excluded.

For the purpose of the review, consensus is defined as electronic communication, an online or in-person meeting, a group teleconference, or the use of a Delphi process among guideline developers. If authors provide little to no detail about the method of consensus used but indicate the use of consensus, it will be included, as long as other methods of development are explicitly described.

Where a reporting guideline has been updated over time and several versions exist, each version will be included as a separate reporting guideline in order to track the number of evaluations of each.

##### *Eligibility of evaluations of reporting guidelines*

Any comparative study with the primary intent of assessing completeness of reporting of at least one study published in a journal that endorses reporting guidelines will be included and considered an “evaluation” for the remainder; studies contained within an evaluation will be termed “studies”. For the first comparison, evaluations using before-after designs to compare completeness of reporting of studies published pre- and post-endorsement within a given journal, or cohort of journals, will be included. For the second comparison, evaluations using a cross-sectional design to compare completeness of reporting between studies published in endorsing and non-endorsing journals will be included. Evaluations examining both types of comparisons will be included.

An editorial statement regarding the use of one or more reporting guidelines is the minimum criterion for a journal to be considered an “endorser”, implying that, at least in principle, the guideline(s) is/are incorporated into the journal’s editorial process. When included evaluations do not report the endorsement status of journals publishing evaluated studies (that is, it is unclear whether studies being evaluated were published in endorsing or non-endorsing journals), corresponding authors will be contacted up to two times to provide this information. If authors did not collect this information, they will be asked to provide the list of studies or journals included in their evaluation; if feasible, members of the review team will seek out the endorsement status of included journals’ from “Instructions to Authors” on their websites. If not feasible to follow-up on an author’s list of studies or journals to obtain information on endorsement status, the evaluation will be excluded with the reason provided. Further to endorsement status, the date of a journal’s endorsement of a reporting guideline is important to ascertain and will help determine whether studies in a given journal were published after a sufficient period of time to allow for changes in editorial policies to be realized in its publication output. For this review, six months will be considered a reasonable period. As it is unlikely that the date of journal endorsement will be reported in evaluations, if feasible, corresponding authors of evaluations or editors of evaluated journals will be contacted for this information. If unavailable, evaluations will not be excluded on this basis; the most recent endorsement status will be used as a surrogate.

Evaluations using interrupted time series designs will be excluded.

Since the CONSORT Statement is a known reporting guideline and a systematic review of its evaluations has recently been updated [20], data from eligible studies will be included in this review. The search strategy of this review intends to identify any CONSORT evaluations published since the date of the last search of that systematic review (March 2010).

### Types of outcomes

#### *Primary outcome*

The primary outcome will be completeness of reporting, This will be measured as adequate or inadequate reporting of any of the following surrogate outcomes, as judged and reported in included evaluations: one or more items in a reporting guideline checklist; a “summary score” of some or all checklist items; a flow diagram (if not already included as a checklist item); or narrative guidance found in the text.

#### *Secondary outcomes*

1. Methodological/reporting quality of an evaluation’s included studies, as measured by any means in the evaluation (for example, Jadad scale for randomized controlled trials).

2. Any unwanted effect described as associated with use of a reporting guideline (for example, increased word count).

### Methods for searching for studies

#### *Identifying reporting guidelines of interest*

Reporting guidelines included in the Moher 2011 systematic review [8], identified through a search ending in September 2009, will be automatically included in this review; guidelines were selected using the same eligibility criteria. From October 2009 to June 2011, reporting guidelines identified by the EQUATOR Network through a comprehensive PubMed search (see Additional file 1: Appendix 1 for full strategy) will be screened for potential inclusion (that is, screened using the above-listed criteria).

#### *Identifying evaluations of reporting guidelines*

A comprehensive two-phase approach has been developed to identify evaluations of reporting guidelines.

1. Some existing reporting guidelines are formally named using a unique acronym in their title (for example, PRISMA: Preferred Reporting Items for Systematic reviews and Meta-Analyses). As well, some reporting guidelines have been widely disseminated across multiple journals or are accompanied by explanatory documents (for example, CONSORT). To capture evaluations for such guidelines, a strategy to identify evaluations referring to any publication of a uniquely named reporting guideline was developed. MEDLINE, EMBASE, and the Cochrane Methodology Register will be searched from 1990 onward using a search strategy that includes the unique acronym of each reporting guideline (see MEDLINE strategy in Additional file 2: Appendix 2). This search strategy is largely based on that used to identify evaluations of the CONSORT Statement in the aforementioned CONSORT systematic review [20].

2. For other reporting guidelines with commonly used acronyms or those without acronyms, a different approach has been developed. For instance, “TREND” refers to guidance on the Transparent Reporting of Evaluations with Nonrandomized Designs but may also refer to statistical trends. Potential evaluations of such guidelines will be identified through forward citation searching of each instance of a guideline’s publication in the Web citation index, Scopus. For those guidelines, MEDLINE and EMBASE will be searched to identify additional, existing multiple publications to search in Scopus.

When a reporting guideline has been updated over time and several versions exist (for example, 1996, 2001 and 2010 versions of CONSORT), potential evaluations of all versions will be accounted for in the search. Reporting guideline developers will also be contacted to ascertain knowledge of any unpublished or in-progress evaluations. Reference lists of related systematic reviews encountered during the screening process will also be hand-searched to identify additional evaluations. Any evaluations detected by members of our team, publically available before the date of the last search will be considered for inclusion.

Published and unpublished evaluations will be included. Letters, comments and editorial publication types will be excluded. There will be no language restriction on the search strategy, but only those available in English and French will be included, due to limited time and resources. Potential evaluations identified in other languages will be listed and set aside for consideration at a future date. Electronic searches were peer reviewed using the Peer Review of Electronic Search Strategies (PRESS) Statement [24]; see Additional file 3: Appendix 3 for peer review comments.

## Data collection and analysis

### Data management

Following the execution of all searches, the identified records (titles and/or available abstracts) will be collated in a Reference Manager® [25] database for de-duplication. The final unique record set and full-text of potentially eligible studies will be exported to an Internet-based software, DistillerSR® (Evidence Partners, Ottawa, ON, Canada), through which screening of records will be carried out. Extraction of data from studies will be carried out in Microsoft Excel (2007, Microsoft Corporation, Redmond, WA, USA).

### Study selection

#### *Selecting reporting guidelines of interest*

Two members of the research team will independently apply inclusion criteria to full text reports of potential reporting guidelines. Screening forms will be piloted using a subset of records. Discordance between reviewers will be resolved through consensus or by a third team member.

#### *Selecting evaluations of reporting guidelines*

The results of the literature search will be assessed using a two-step process:

1. One individual will screen citations by titles and/or abstract according to the pre-specified screening questions (level 1). For those records deemed to be ‘included’ and/or ‘unclear’, they will automatically pass to the next level of screening (level 2). However, if the record is deemed ‘excluded’, then it will be assessed by a second reviewer to confirm exclusion. This process is referred to as ‘liberal accelerated’ screening, a more efficient means of initially assessing records for relevancy.

2. Full-text screening will be conducted by two independent reviewers over two phases, where pre-defined questions will be split to create two levels (levels 2 and 3). This will be done in order to expedite the screening process - eligibility based on some criteria can be determined simply from the evaluation report whereas other criteria may require contact with corresponding authors of evaluations in order to judge eligibility (for example, endorsement status of journals in the evaluations). Discordance between reviewers will be resolved through consensus or by a third team member.

3. All screening forms will be piloted using a subset of records. Screeners will not be blinded to study authors or journal of publication.

### Data extraction

Separate data extraction forms will be developed to capture information needed for synthesis for each of the two comparisons of this review and will be piloted using a subset of included evaluations and modified, as needed. One reviewer will extract general study characteristics of included evaluations, with verification of a random 10% of studies carried out by a second reviewer. Data on completeness of reporting for each reporting guideline will be extracted by one reviewer; a second reviewer will verify the accuracy of the data from a random 20% sample of included evaluations. Any discrepancies between reviewers will be resolved by consensus or by a third member of the research team. If there are greater than 50% discrepancies, 100% data verification will be considered.

Data items that will be extracted from evaluations will include:

Name of reporting guideline being evaluated (with version if applicable)

Whether an included reporting guideline is an extension of the primary reporting guideline. If so, whether it is an official extension (that is, developed in collaboration with primary guideline authors) will be determined. Collaboration will be defined as inclusion of at least one lead author of the original guideline on the extension authorship list.

Study design of the evaluation (for example, cross-sectional, cohort, and so on).

General characteristics of the evaluation: first author name; year of publication; country of corresponding author; corresponding author email address; source of funding.

Characteristics of studies assessed in the evaluation: number of studies and publishing journals; date of study publication; journal endorsement status and date of journal endorsement (if available); whether date of endorsement was determined by evaluation authors at the time of evaluation, obtained directly from journals, or determined by review authors (based on current/surrogate status listed in journals’ “Instructions to Authors”; extent of endorsement according to pre-defined categories (see ‘Subgroup analysis’ section); medical specialty; guideline checklist items assessed, if applicable.

Completeness of reporting will be collected as measured by any of the following surrogate outcomes:

Adequacy of reporting of individual checklist items or a combination of items into a summary score.

It is anticipated that most studies will evaluate completeness of reporting in this manner, since 94% of reporting guidelines identified in a recent systematic review included a checklist [9].

Item-by-item extraction forms will be developed for each checklist-based reporting guideline that is assessed in at least one evaluation.

Where evaluations present data on variations of checklist items, this data will be collected and presented in subgroup analyses (see ‘Subgroup analysis’ section).

Calculation of a summary score may be misleading since items within a checklist are not necessarily of equal importance. However, if completeness of reporting is assessed in this manner in included evaluations, data will be collected and analyzed.

Adequacy of reporting according to narrative guidance found in the text of a guideline document.

For evaluations of reporting guidelines without a checklist, methods of evaluation of completeness of reporting and overall completeness of reporting, as reported, will be collected.

Adequacy of reporting other measures of completeness of reporting, if assessed in evaluations.

Other measures of study quality reported in the evaluation, however measured (for example, Jadad scale for randomized controlled trials).

Potential unwanted effects from using a reporting guideline, as reported by authors of an included evaluation, however reported.

Authors of potentially included evaluations will be contacted up to two times to obtain additional information, if needed, such as outcome data or data not available in published reports.

### Assessment of validity of included studies

One reviewer will independently assess internal validity for each included evaluation; this will be verified by a second reviewer. A set of criteria has been developed for this review, based on concepts presented in the Data Collection Checklist developed by the Cochrane Effective Practice and Organisation of Care Review Group [26], the Strengthening the Reporting of Observational Studies in Epidemiology (STROBE) Statement [27], the Newcastle-Ottawa Quality Assessment Scale [28] and the Cochrane Risk of Bias Tool [29].

#### *Criteria for assessing validity*

1. Whether more than one person evaluated completeness of reporting of included studies

2. Whether the set of items to be evaluated (methods) is the same as those which were evaluated (results)

3. Whether the intended set of data is completely reported or provided

4. Whether the search strategy used to identify studies in the evaluation was appropriate to the question being asked

5. Whether included studies have a particular publication frequency within included journals

6. If no to the item 5, whether confounding considered/accounted for in the evaluation

Each criterion will be judged as yes (high validity), no (low validity), unclear (not reported) and not applicable; support for judgments will also be provided. As no methods exist for synthesizing these data into a summary judgment, results of validity assessment will be presented in tabular format for reader interpretation.

### Measures of effect

The first comparison is the completeness of reporting within endorsing journals before and after endorsement, and the second is completeness of reporting between endorsing and non-endorsing journals in the same period. If data for both comparisons are available in a single evaluation, the evaluation will be included in both comparisons (that is studies are published in endorsing and non-endorsing journals and the endorsing cohort includes the time periods before and after endorsement).

Within each evaluation, the proportion of studies adequately reporting one or more checklist items for a given guideline will be collected. For guidelines containing only text-based recommendations, if evaluations exist, recommendations will be grouped into meaningful pseudo-checklist items and adequacy of reported data will be collected for each item. A relative risk (RR) and 99% confidence intervals will be calculated for each study; a 99% confidence interval will ensure conservative estimates of precision are obtained. A RR > 1.0 will indicate a higher proportion of studies adequately reporting a given checklist item.

Likewise, means and standard deviations (SDs) will be collected for checklist items combined into a summary score; when medians and ranges are reported instead of means and SDs, suitable approximations will be used, as discussed in the Cochrane Handbook [30]. A standardized mean difference (SMD) and 99% confidence interval will be calculated for each study; an SMD>0 will indicate more adequate reporting of checklist items contained within the total summary score.

Information on the methodological quality of studies within evaluations and any unwanted effects of reporting guideline use will be presented as reported in the evaluations. No judgments will be made on these items; they will be presented as collected from included evaluations.

### Unit of analysis

The unit of analysis in this review is an included evaluation. Within each evaluation, one or more included studies may be published in the same journal, thereby not independent due to a common effect from editorial policies. As such, assessment of this issue will be carried out during validity assessment of evaluations, as described above (see validity items 5 and 6).

### Dealing with missing data

Corresponding authors of potentially included evaluations will be contacted, up to two times, where data are needed (that is endorsement status of include journals, completeness of reporting assessments). If the data are not obtained and compromise the ability to include the evaluation in quantitative synthesis, they will be excluded from the meta-analyses.

### Reporting Biases

Asymmetry of funnel plots is an established method for assessing the potential presence of publication bias in traditional systematic reviews of intervention effectiveness, subject to a sufficient number of included studies [30,31]. Funnel plots are a graphical representation of individual study estimates of effect against a measure of the study’s size or precision. In the current study, the sample size is the number of studies included in each evaluation. Although it is possible to generate funnel plots to assess the potential of publication bias within our pool of included evaluations, both the suitability and possible interpretation of such plots are unknown.

### Data synthesis

For each included evaluation we will present study characteristics, assessment of validity, and description of the reporting guideline evaluations in a series of tables and a narrative summary. Meta-analyses will be carried out using the Review Manager [31] and Comprehensive Meta-analysis software [32]. If evaluations for each reporting guideline are similar enough on the basis of study design, for each outcome, effect estimates from each evaluation will be pooled into a single, overall, effect estimate.

#### Primary outcome

Pooled RRs and MDs with corresponding 99% confidence intervals, using a random effects model, will be used to compare completeness of reporting of studies across evaluations for each checklist item or summary score, respectively, for each guideline. Estimates of effect for different reporting guidelines will not be pooled in any way; subgroups and totals will be provided for each guideline, including various versions of a given reporting guideline, separately. Data from guidelines with only text-based recommendations will not be pooled due to anticipated variation in how adequacy of reporting data was collected.

#### *Secondary outcomes*

It is expected that methods of assessment of methodological quality and reporting of unwanted effects will be variable among included evaluations. When reported, a descriptive summary of methodological quality of studies included in evaluations and unwanted effects, for each reporting guideline, will be provided. No attempt will be made to statistically synthesize these data.

### Subgroup analysis

The following subgroup analyses are planned, if feasible:

*Extent of reporting guideline endorsement by journals included in evaluations:* pre-defined groupings developed by authors of the recent CONSORT review will be used [20]: (a) any editorial statement regarding use of a guideline; (b) recommendation in a journal’s “Instructions to Authors” to follow the guideline when preparing the manuscript; and (c) requirement for authors to submit guideline adherence documentation (for example, completed CONSORT checklist) with their manuscript.

*Variations in checklist items*: if variations in how evaluations report completeness of reporting for different checklist items are encountered, data will be presented in the main analyses according to different subgroups for each variation. For example, in the CONSORT systematic review, ‘blinding’ was reported in four different ways among evaluations and, therefore, data divided into four subgroups, accordingly, in the analysis [20].

*Official and unofficial extensions of reporting guidelines:* for reporting guidelines identified as extensions to a primary guideline, effect estimates for official and unofficial extensions will be presented separately.

### Sensitivity analyses

We plan to conduct the following sensitivity analyses, if possible, to determine the influence on effect estimates:

Six-month endorsement period. The primary outcome analysis will be restricted to evaluations of studies that were published at least six months following the date of journal endorsement, for which the true date of endorsement could be obtained (that is, as provided in the report, by evaluation authors or by journal editors, not the date on which surrogate status was obtained by review authors).

Study outliers. Evaluations with effect estimates outside of the 99% CI of pooled RRs and SMDs will be removed for sensitivity analysis.

### Assessment of heterogeneity

We plan to measure the inconsistency of study results using the I^2^ heterogeneity statistic to determine the extent of variation in effect estimates that is due to heterogeneity rather than chance. Heterogeneity, as defined by Higgins, is measured as a percentage (%) where a value ≤25% for I^2^ indicates low heterogeneity, 26% to 50% indicates moderate heterogeneity 51% to 75% indicates substantial heterogeneity and 76% to 100% indicates considerable heterogeneity [30]. Substantially heterogeneous effect estimates will not be pooled. Possible reasons for heterogeneity will be explored in sensitivity analyses; the pre-specified subgroup analyses, if feasible, will be examined to determine whether they provide possible reasons for any observed statistical heterogeneity.

### Reporting of this review

This systematic review will be reported according to the Preferred Reporting Items for Systematic reviews and Meta-Analyses (PRISMA) Statement [33] – a reporting guideline for systematic reviews of healthcare interventions – and will include a PRISMA checklist. Where necessary, we will adapt the reporting to ensure that all items relevant to this review are included in the report.

## Discussion

This systematic review aims to provide evidence to help guide decision making for journal editors and publishers. While some editors are enthusiastic about reporting guidelines, it is likely a prudent policy to endorse and adhere to those reporting guidelines that are appropriately developed and provide some evidence of effectiveness; namely, that their use is associated with improved completeness of reporting. The proposed systematic review will provide the evidence regarding reporting guideline effectiveness. As such we believe that we are providing editors with evidence to help inform their prospective policy about specific reporting guidelines.

Beyond journal editors we believe that the results of this review will be of relevance to other knowledge users, namely, peer reviewers and authors. There is an increased interest in asking peer reviewers to use reporting guidelines as part of their assessment of manuscripts; 46% of health journals surveyed (n = 116) mentioned reporting guidelines in their instructions to peer reviewers [34]. The results of this review will help peer reviewers decide which reporting guidelines are effective and, thus, likely more beneficial to use as part of the peer review process. Finally, authors should be more strongly encouraged to use reporting guidelines for which there is evidence that they are associated with improved completeness of reporting.

With information summarized in this review, publishers may be able to implement reporting guidelines across a wide spectrum of journals and reduce the burden on individual journals. Specifically, making reporting guidelines accessible and required at the time of submission will greatly improve their use by authors and ease the burden of peer-reviewing by standardizing the selection criteria across journals, specific to the research typology being reviewed.

## Abbreviations

EQUATOR: Enhancing the Quallity and Transparency of health Research; CONSORT: Consolidated Standards of Reporting Trials; STARD: Standards for the Reporting of Diagnostic Accuracy; STRICTA: Standards for Reporting Interventions in Clinical Trials of Acupuncture; PRISMA: Preferred Reporting Items for Systematic reviews and Meta-Analyses; PRESS: Peer Review of Electronic Search Strategies; STROBE: Strengthening the Reporting of Observational Studies in Epidemiology; RR: Relative risk; SMD: Standardized mean difference.

## Competing interests

Professor Altman, and Drs. Hoey, Moher and Schulz are executive members of the EQUATOR network; Dr. Iveta Simera and Allison Hirst are EQUATOR staff members. The EQUATOR Network is funded by the National Health Service (NHS) National Library of Health, NHS National Institute for Health Research, NHS National Knowledge service, UK Medical Research Council, Canadian Institutes of Health Research, Scottish Chief Scientist Office, Pan American Health Organization.

This study is supported by a grant from the Canadian Institutes of Health Research (#234489). CIHR had no role in study design, plans for data collection and analysis, decision to publish, or preparation of this protocol.

Professor Altman is supported by Cancer Research UK, Dr. Moher by a University of Ottawa Research Chair, and Dr. Schulz by FHI360. All researchers are independent from their relevant funding agencies.

## Authors contributions

LS, AS and DM have made substantial contributions to conception, design and preparing the first draft of this protocol. All authors have contributed to revising this protocol critically for important intellectual content and have given final approval of the version to be published. All authors read and approved the final manuscript.

## Supplementary Material

Additional file 1 **Appendix 1.** EQUATOR network Pubmed Reporting Guidelines Search Strategy. Click here for file

Additional file 2 **Appendix 2.** MEDLINE search strategy for evaluations of reporting guidelines with acronyms. Searches were tailored to search EMBASE and the Cochrane Methodology Register. Searches for remaining reporting guidelines were conducted in Scopus. Click here for file

Additional file 3 **Appendix 3.** PRESS EBC Search Submission. Click here for file
