# Supplementary material for: Does journal endorsement of reporting guidelines influence the completeness of reporting of health research? A systematic review protocol
Source: Syst Rev. 2012 May 24;1:24. doi: 10.1186/2046-4053-1-24 (PMC3482392; doi:10.1186/2046-4053-1-24)
Supplement: Additional file 1 — Appendix 1. EQUATOR network Pubmed Reporting Guidelines Search Strategy. [file 2046-4053-1-24-S1.docx]

Appendix 1

EQUATOR network Pubmed Reporting Guidelines Search Strategy

1. reporting guideline* [tiab]

2. guideline* [ti] AND reporting [ti]

3. guidance [tiab] AND reporting [tiab]

4. reporting requirement* [tiab]

5. guideline* [ti] AND publication* [ti]

6. standard* [ti] AND reporting [ti]

7. practice [ti] AND reporting [ti]

8. design [ti] AND reporting [ti]

9. conduct [ti] AND reporting [ti]

10. reporting [ti] AND criteri* [ti]

11. reporting [ti] AND recommendation* [ti]

12. research reporting [tiab]

13. transparen* [tiab] AND reporting [tiab]

14. responsible [ti] AND report* [ti]

15. clarity [ti] AND report* [ti]

16. presentation [ti] AND publication [ti]

17. analys* [ti] AND reporting [ti]

18. presentation [ti] AND standard* [ti]

19. presentation [ti] AND guideline* [ti]

20. minimum information [tiab]

21. reporting [ti] AND method* [ti]

22. reporting [ti] AND experiment* [ti]

23. #1 or #2 or #3 or #4 or #5 or #6 or #7 or #8 or #9 or #10 or #11 or #12 or #13 or #14 or #15 or #16 or #17 or #18 or #19 or #20 or #21 or #22

24. Guidelines as Topic [Mesh]

25. Practice Guidelines as Topic [Mesh]

26. Randomized Controlled Trials as Topic/standards [Mesh]

27. Randomized Controlled Trials as Topic/methods [Mesh]

28. Randomized Controlled Trials as Topic/statistics and numerical data [Mesh]

29. Clinical Trials as Topic/standards [Mesh]

30. Clinical Trials as Topic/methods [Mesh]

31. Clinical Trials as Topic/statistics and numerical data [Mesh]

32. Meta-Analysis as Topic [Mesh]

33. Periodicals as Topic [Mesh]

34. Research Design [Mesh]

35. Guideline Adherence [Mesh]

36. Consensus [Mesh]

37. Publishing/standards [Mesh]

38. Publications/standards [Mesh]

39. Writing/standards [Mesh]

40. Evidence-Based Practice/standards [Mesh]

41. Evidence-Based Practice/methods [Mesh]

42. Evidence-Based Medicine [Mesh]

43. Sensitivity and Specificity [Mesh]

44. Reproducibility of results [Mesh]

45. Biomedical Research/methods [Mesh]

46. Biomedical Research/standards [Mesh]

47. Quality Control [Mesh]

48. Editorial policies [Mesh]

49. Health Services Research/standards [Mesh]

50. Health Services Research/methods [Mesh]

51. Epidemiologic studies [Mesh]

52. Authorship [Mesh]

53. Case Control Studies [Mesh]

54. Biomedical Research [Mesh]

55. Data Interpretation, Statistical [Mesh]

56. Technology Assessment, Biomedical/statistics and numerical data [Mesh]

57. Review [pt]

58. Meta-analysis [pt]

59. Editorial [pt]

60. Clinical Trial [pt]

61. Guideline [pt]

62. Case Reports [pt]

63. #24 or #25 or #26 or #27 or #28 or #29 or #30 or #31 or #32 or #33 or #34 or #35 or #36 or #37 or #38 or #39 or #40 or #41 or #42 or #43 or #44 or #45 or #46 or #47 or #48 or #49 or #50 or #51 or #52 or #53 or #54 or #55 or #56 or #57 or #58 or #59 or #60 or #61 or #62

64. report* [ti] AND quality [ti]

65. reporting checklist* [tiab] OR reporting statement* [tiab]

66. report* [tiab] AND instruction* [tiab]

67. reporting [tiab] AND policy [tiab]

68. reporting [tiab] AND policies [tiab]

69. reporting parameter* [tiab] OR adverse event report* [tiab]

70. research standard [tiab] OR research standards [tiab]

71. standardized reporting [tiab] OR standardised reporting [tiab]

72. current reporting [tiab]

73. reporting [ti] AND stud* [ti]

74. reporting [ti] AND trial* [ti]

75. minimum quality level [tiab]

76. standards [tiab] AND reporting [tiab]

77. reporting [ti] AND result* [ti]

78. reporting [ti] AND data [ti]

79. reporting [ti] AND statistic* [ti]

80. responsible [ti] AND research [ti]

81. accura* [ti] AND reporting [ti]

82. uniform* [ti] AND guideline* [ti]

83. uniform* [ti] AND report* [ti]

84. author* [ti] AND guideline* [ti]

85. advice [ti] AND author* [ti]

86. journal* [tiab] AND submit* [ti]

87. journal* [tiab] AND submission [ti]

88. appropriate* [ti] AND report* [ti]

89. reporting [ti] AND research [ti]

90. guidelines [ti] AND authors [ti]

91. contributor* [tiab] AND journal* [tiab]

92. publication* [ti] AND data [ti]

93. recommendation* [tiab] AND editor* [tiab]

94. author* [ti] AND instruction* [tiab]

95. publication* [ti] AND practice* [ti]

96. CONSORT [tiab] OR PRISMA [tiab] OR STARD [tiab] OR STRICTA [tiab] OR REDHOT [tiab] OR GNOSIS [tiab] OR REHBaR [tiab] OR STROBE [tiab] OR STREGA [tiab] OR GRRAS [tiab] OR COREQ [tiab] OR SQUIRE [tiab] OR ARRIVE [ti] OR ORION [ti] OR REMARK [ti] OR Utstein [ti] OR IUPAC recommendation* [tiab] OR GRIPS statement [tiab] OR STARE-HI [tiab]

97. #64 or #65 or #66 or #67 or #68 or #69 or #70 or #71 or #72 or #73 or #74 or #75 or #76 or #77 or #78 or #79 or #80 or #81 or #82 or #83 or #84 or #85 or #86 or #87 or #88 or #89 or #90 or #91 or #92 or #93 or #94 or #95 or #96

98. #63 and #97

99. #23 or #98
