# Supplementary material for: Does journal endorsement of reporting guidelines influence the completeness of reporting of health research? A systematic review protocol
Source: Syst Rev. 2012 May 24;1:24. doi: 10.1186/2046-4053-1-24 (PMC3482392; doi:10.1186/2046-4053-1-24)
Supplement: Additional file 2 — Appendix 2. MEDLINE search strategy for evaluations of reporting guidelines with acronyms. Searches were tailored to search EMBASE and the Cochrane Methodology Register. Searches for remaining reporting guidelines were conducted in Scopus. [file 2046-4053-1-24-S2.docx]

Appendix 2

MEDLINE search strategy for evaluations of reporting guidelines with acronyms. Searches were tailored to search EMBASE and the Cochrane Methodology Register. Searches for remaining reporting guidelines were conducted in Scopus.

1 "Meta-analysis Of Observational Studies in Epidemiology".ti,ab.

2 MOOSE.ti,ab.

3 limit 2 to animal

4 2 not 3

5 1 or 4

6 ((standard$1 adj2 "reporting of diagnostic accuracy") or STARD).ti,ab.

7 ("Consolidated Standards of Reporting Trials" or CONSORT).ti,ab.

8 ("Standards for Reporting Interventions in Controlled Trials of Acupuncture" or STRICTA).ti,ab.

9 ("Reporting data on homeopathic treatments" or RedHot).ti,ab.

10 ("Statement on reporting of evaluation studies in Health Informatics" or "STARE-HI").ti,ab.

11 ("Minimum Information Required for Reporting a Molecular Interaction Experiment" or MIMIX).ti,ab.

12 ("Minimum Information Specification for In Situ Hybridization" or MISFISHIE).ti,ab.

13 ("Minimum Information about a Proteomics Experiment" or MIAPE or MIAPEMS or MIAPE-MS).ti,ab.

14 ("Strengthening the Reporting of Genetic Association Studies" or STREGA).ti,ab.

15 ("Strengthening the Reporting of Observational Studies in Epidemiology" or STROBE).ti,ab.

16 ("Outbreak Reports and Intervention Studies of Nosocomial Infection" or ORION).ti,ab.

17 ("Standards for Quality Improvement Reporting Excellence" or SQUIRE).ti,ab.

18 ("Completeness of reporting of Meta-Analyses" or "Completeness of reporting of Metaanalyses" or "Completeness of reporting of Metanalyses" or (QUORUM adj5 (reporting or meta-analy* or metaanaly* or metanaly* or systematic review* or statement* or guideline* or checklist* or criteria* or flowchart* or flow chart* or flow diagram*))).ti,ab.

19 ("Preferred Reporting Items for Systematic Reviews and Metaanalyses" or PRISMA).ti,ab.

20 ("Strengthening the reporting of genetic risk prediction studies" or GRIPS).ti,ab.

21 ("Reporting Experiments in Homeopathic Basic Research" or REHBaR).ti,ab.

22 ("Guidelines for Reporting Reliability and Agreement Studies" or GRRAS).ti,ab.

23 ("Standard guidelines for publication of deep brain stimulation studies" or "Guide4DBS-PD").ti,ab.

24 (good publication practice$1 or GPP2).ti,ab.

25 "Utstein style".ti,ab.

26 or/5-25

27 limit 26 to (comment or editorial or guideline or letter)

28 26 not 27

29 limit 28 to yr="1990-Current"
