# Supplementary material for: Does journal endorsement of reporting guidelines influence the completeness of reporting of health research? A systematic review protocol
Source: Syst Rev. 2012 May 24;1:24. doi: 10.1186/2046-4053-1-24 (PMC3482392; doi:10.1186/2046-4053-1-24)
Supplement: Additional file 3 — Appendix 3. PRESS EBC Search Submission. [file 2046-4053-1-24-S3.pdf]

PRESS EBC Search Submission

Searcher's Name: Becky Skidmore **E-mail:** bskidmore@rogers.com

Date submitted: 14 Oct 2011 **Date needed by:** ASAP

Note to peer reviewers – please enter your information in the Peer Review Assessment area

Remember: this peer review only pertains to your MEDLINE search strategy.

Search question (Describe the purpose of the search)

Our review will employ conventional systematic review methods targeting 81 reporting guidelines identified from a recent systematic review. Our search strategy will identify evaluations of these guidelines. We will also compare characteristics of guidelines and journals that are associated with usage. Analysis methods will follow those used in a systematic review of evaluations of the CONSORT 2001 Statement.

PICO format (Outline the PICO for your question, i.e., the Patient, Intervention, Comparison and Outcome)

P: *Doesn't really apply*

I:

C:

O:

Inclusion criteria (List any inclusion criteria, such as age groups, study designs, to be included)
See attached list of Reporting Guidelines

Exclusion criteria (List any exclusion criteria, such as study designs, to be excluded)

Was a search filter applied? (Remember this pertains only to the MEDLINE strategy)

Yes ☐ *No* ☒

If yes, which one?

Cochrane hedge:
Haynes/McKibbon et al:
CRD (UK):
Other:
PUBMED clinical query:
SIGN (Scottish):
Robinson and Dickerson:

MEDLINE search interface used

EBSCO ☐ *OVID* ☒ *PubMED* ☐ *Other* ____________

Has the search strategy been adapted (i.e., subject heading and terms reviewed) for other databases? Please check all that apply.

Ageline	☐
AMED	☐
C2-SPCTRE	☐
CINAHL	☐
Cochrane Database of Systematic Reviews (CDSR; Cochrane Reviews)	☐
Cochrane Central Register of Controlled Trials (CENTRAL; Clinical Trials)	☐
Cochrane Methodology Register (CMR; Methods Studies)	☒
Cochrane Library (all databases)	☐
Database of Abstracts of Reviews of Effects (DARE; Other Reviews)	☐
Embase	☒
ERIC	☐
LILACS (Latin American and Caribbean Health Sciences Literature)	☐
MEDLINE	☒
PsycINFO	☐
PreMEDLINE	☐
Other	☐
Other	☐
Other	☐

Other notes or comments that you feel would be useful for the peer reviewer?

Date limit 1990 – current

We are looking for items that have done any sort of evaluation or impact study since the creation/implementation of the guideline. The rationale has changed such that we will do an OVID and CMR search for the named reporting guidelines provided the acronym does not generate too much irrelevant material. This has meant that items such as TREND, REMARK, ROBUST, etc. will only be searched in the forward citation search. The exceptions are CONSORT & QUORUM. All CONSORT extensions will be searched in Web of Science in a forward citation fashion – the "original", since it was published in multiple journals and has been cited so excessively, is being searched in OVID only and it is anticipated that many of the extensions will be picked up that way, too. QUORUM will be searched in both – in order to control #s in OVID some additional vocabulary has been added for extra precision (maybe I should also add flowchart$1, flow chart$1, flow diagram$1 ??).

Please paste your MEDLINE strategy here:

Database: Ovid MEDLINE(R) In-Process & Other Non-Indexed Citations and Ovid MEDLINE(R) <1948 to Present> Search Strategy:

----

1 "Meta-analysis Of Observational Studies in Epidemiology".ti,ab. (34)
2 MOOSE.ti,ab. (601)
3 limit 2 to animal (436)
4 2 not 3 (165)
5 1 or 4 (172)
6 ((standard$1 adj2 "reporting of diagnostic accuracy") or STARD).ti,ab. (374)
7 ("Consolidated Standards of Reporting Trials" or CONSORT).ti,ab. (895)
8 ("Standards for Reporting Interventions in Controlled Trials of Acupuncture" or STRICTA).ti,ab. (166)
9 ("Reporting data on homeopathic treatments" or RedHot).ti,ab. (9)
10 ("Statement on reporting of evaluation studies in Health Informatics" or "STARE-HI").ti,ab. (7)
11 ("Minimum Information Required for Reporting a Molecular Interaction Experiment" or MIMIX).ti,ab. (14)
12 ("Minimum Information Specification for In Situ Hybridization" or MISFISHIE).ti,ab. (4)
13 ("Minimum Information about a Proteomics Experiment" or MIAPE or MIAPEMS or MIAPE-MS).ti,ab. (20)
14 ("Strengthening the Reporting of Genetic Association Studies" or STREGA).ti,ab. (14)
15 ("Strengthening the Reporting of Observational Studies in Epidemiology" or STROBE).ti,ab. (331)
16 ("Outbreak Reports and Intervention Studies of Nosocomial Infection" or ORION).ti,ab. (519)
17 ("Standards for Quality Improvement Reporting Excellence" or SQUIRE).ti,ab. (153)
18 ("Quality of Reporting of Meta-Analyses" or "Quality of Reporting of Metaanalyses" or "Quality of Reporting of Metanalyses" or (QUORUM adj5 (reporting or meta-analy* or metaanaly* or metanaly* or systematic review* or statement* or guideline* or checklist* or criteria*))).ti,ab. (79)
19 ("Preferred Reporting Items for Systematic Reviews and Metaanalyses" or PRISMA).ti,ab. (343)
20 ("Strengthening the reporting of genetic risk prediction studies" or GRIPS).ti,ab. (650)
21 ("Reporting Experiments in Homeopathic Basic Research" or REHBaR).ti,ab. (1)
22 ("Guidelines for Reporting Reliability and Agreement Studies" or GRRAS).ti,ab. (4)
23 ("Standard guidelines for publication of deep brain stimulation studies" or "Guide4DBS-PD").ti,ab. (1)
24 (good publication practice$1 or GPP2).ti,ab. (50)
25 "Utstein style".ti,ab. (208)
26 or/5-25 (3898)
27 limit 26 to (comment or editorial or guideline or letter) (349)
28 26 not 27 (3549)
29 limit 28 to yr="1990-Current" (3050)

© Sampson M, McGowan J, Lefebvre C

Peer Review Assessment
[For peer reviewers only]

Peer reviewer's name: Andra Morrison

Press #:

E-mail: andra_morrison@hotmail.com; andram@cadth.ca

Date completed: 16 Oct 2011

Please select the one most appropriate answer for each element

	Adequate	Adequate with revisions*	Needs revision*
1. Translation of the research question	X		
2. Boolean and proximity operators	X		
3. Subject headings	None used		
4. Natural language / free-text	X		
5. Spelling, syntax and line numbers	X		
6. Limits and filters	X		
7. Search strategy adaptations	X		

* Provide an explanation or example for "Adequate with revisions" and "needs revision":

Other Comments (please limit to 3-5 sentences):

On line 18 of the Medline and Embase search, and line 14 of the Cochrane search, it might be worth while to use the term "flowchart" or flow diagram".

I would consider searching for reviews.

I am not sure how you can improve specificity with some of these titles. With methodological search strategies, I generally consider "noise" to be an assurance that relevant articles are not being missed.

This is a well constructed search strategy.

From: andra morrison [mailto:andra_morrison@hotmail.com]
Sent: Sunday, October 16, 2011 6:38 PM
To: bskidmore@rogers.com
Subject: RE: Reporting Guidelines
Actually, i was refering to searching the CDSR.

From: bskidmore@rogers.com
To: andra_morrison@hotmail.com
Subject: RE: Reporting Guidelines
Date: Sun, 16 Oct 2011 15:55:46 -0400

© Sampson M, McGowan J, Lefebvre C

Thanks, Andra – just want to make sure I get the green light as they are definitely eager to get this going. When you say "I would consider searching for reviews" do you meant creating a search using controlled vocabulary and text words (which is similar to the original strategy) and then applying a review filter?

Becky

From: andra morrison [mailto:andra_morrison@hotmail.com]
Sent: Sunday, October 16, 2011 1:42 PM
To: bskidmore@rogers.com
Subject: RE: Reporting Guidelines

Hi Becky,
I have reviewed your strategy and attached my comments. This was a very dissatisfying (sp?) search strategy to peer review b/c there was absolutely nothing I could fault or improve upon (and believe me, I tried).
Andra

© Sampson M, McGowan J, Lefebvre C
